# Liver Involvement in Children with COVID-19 and Multisystem Inflammatory Syndrome: A Single-Center Bulgarian Observational Study

**DOI:** 10.3390/microorganisms9091958

**Published:** 2021-09-15

**Authors:** Snezhina Lazova, Tea Alexandrova, Nadzhie Gorelyova-Stefanova, Kalin Atanasov, Iren Tzotcheva, Tsvetelina Velikova

**Affiliations:** 1 Pediatric Department, University Hospital “N. I. Pirogov”, General Eduard I. Totleben Blvd. No. 21, 1606 Sofia, Bulgaria; snejina@lazova.com (S.L.); tea_alexandrova@yahoo.com (T.A.); nadzhie.gorelyova@gmail.com (N.G.-S.); kaatanasov23@gmail.com (K.A.); irenmd@yahoo.com (I.T.); 2 Health Care Department, Faculty of Public Health, Medical University Sofia, Bialo More, 8 Str., 1527 Sofia, Bulgaria; 3 Department of Clinical Immunology, Medical Faculty, University Hospital Lozenetz, Sofia University, St. Kliment Ohridski, Kozyak 1 Str., 1407 Sofia, Bulgaria

**Keywords:** multisystem inflammatory syndrome in children, COVID-19, SARS-CoV-2, IL-6, liver, pediatric COVID-19

## Abstract

SARS-CoV-2 infection may precede and cause various autoimmune and inflammatory diseases, including multisystem inflammatory syndrome in children (MIS-C). Therefore, we aimed to observe the clinical presentation and laboratory, instrumental and other constellations in children with MIS-C, including liver involvement. We present the outcomes from a single-center prospective observational study in which 89 children was included (60 with proven COVID-19, 10 symptomatic with confirmed COVID-19 contact and 19 diagnosed with MIS-C). Laboratory, instrumental, immunological, and clinical investigations were performed. Only 12% (*n* = 4) from the COVID-19 group (except the ICU cases), we found elevated AST and/or ALT (up to 100). All of the children with elevated transaminase were overweight or obese, presenting along with moderate COVID-19 pneumonia. The majority of children with MIS-C showed typical laboratory constellations with higher levels of IL-6 (120.36 ± 35.56 ng/mL). About half of the children in the MIS-C group (52%, *n* = 11) showed elevated transaminases. Eleven children (57.9%) presented with abdominal pain, eight (42.1%) with ascites, two (10.5%) with hepatosplenomegaly, and four (21.1%) with symptoms such as diarrhea. Mesenteric lymphadenitis was observed more often in patients with elevated LDH (327.83 ± 159.39, *p* = 0.077). Ascites was associated with lymphopenia (0.86 ± 0.80, *p* = 0.029) and elevated LDH. Hepato-splenomegaly was also more frequent in children with lymphopenia (0.5 ± 0.14, *p* = 0.039), higher troponin (402.00 ± 101.23, *p* = 0.004) and low ESR. Diarrhea was more frequent in patients with lower CRP (9.00 ± 3.44 vs. 22.25 ± 2.58, *p* = 0.04), and higher AST and ALT (469.00 ± 349.59 vs. and 286.67 ± 174.91, respectively, *p* = 0.010), and D-dimer (4516.66 ± 715.83, *p* = 0.001). Our data suggest that the liver can also be involved in MIS-C, presenting with typical laboratory and instrumental outcomes.

## 1. Introduction

At the beginning of the pandemic, the SARS-CoV-2 was perceived as a lower respiratory tract infection, affecting the lung parenchyma predominantly and potentially leading to acute respiratory distress syndrome (ARDS) [1]. However, with time, it became evident that COVID-19 can present with wide variability of symptoms, including gastrointestinal, neurologic, cardiovascular, and even multiorgan failure, as part of a severe inflammatory response syndrome (SIRS) [2]. Furthermore, research shows that the wide range of clinical manifestations is linked to the viral tropism to the angiotensin-converting enzyme 2 (ACE2) receptor found on many different cells, including liver and bile-duct epithelial cells [3,4]. 

The distribution of ACE2 receptors in the liver is unusual. The receptor is abundant in the endothelial layer of small blood vessels but not in sinusoidal endothelial cells. Chai et al. reported more robust surface expression of ACE2 in cholangiocytes (59.7%) than in hepatocytes (2.6%). The level of ACE2 expression in cholangiocytes was comparable to that of type 2 alveolar cells in the lungs, implying that the liver may be a possible target for SARS-CoV-2 as well. However, Kupffer cells, T and B lymphocytes tested negative for ACE2 on immunohistochemistry staining [5].

Reports to date indicate that SARS-CoV-2 infection precedes the onset of various autoimmune and inflammatory diseases, including childhood inflammatory multisystem syndrome (PIMS), also classified as a multisystem inflammatory syndrome in children (MIS-C) [6]. This information further complicates the understanding of the course of COVID-19 infection in children and post-infectious immune transformation (alteration; readjustment) in children [7,8].

In children, the first reports of MIS-C changed the reputation of SARS-CoV-2 as an infection that mostly spares children with moderate or even asymptomatic presentation to a potentially fatal one with multiorgan involvement and uncertain outcome. Due to the Kawasaki-like manifestation of MIS-C, most of the reports and studies focus mainly on cardiovascular impairment mechanisms and complications [9,10]. Lately, liver involvement has been included in assessing COVID-19 severity infection or MIS-C presentation and the possibility of using liver enzymes as a prognostic sign for the expected outcome [11].

There is a lack of reports and studies on COVID-19 infection in children with preexisting chronic liver disease. However, in the non-pediatric population, infections are associated with decompensation of cirrhosis and the onset of acute or chronic liver failure. This by itself is a risk factor for a severe course of COVID-19 [12]. In addition, comorbidities in adults such as diabetes, hypertension, and obesity often lead to non-alcoholic hepatosteatosis (Fatty Liver Disease). This condition contributes to complications of SARS-CoV-2 infection [13].

Among other organs involved in MIS-C, such as the heart, kidneys, lungs, gastrointestinal, skin, nervous system, and blood, the liver can also be damaged during COVID-19 infection and MIS-C particularly. Therefore, we aimed to observe the clinical presentation and laboratory, instrumental and other constellations in children with MIS-C and COVID-19 infection, focusing on liver involvement.

## 2. Materials and Methods

### 2.1. Study Design and Subjects

We present the outcomes from a prospective observational study in which 89 children with suspected COVID-19 were administered to our pediatric emergency triage and clinic for a period of five months (October 2020 to February 2021). Thirty-nine cases (39) were managed ambulatory (Outpatients), whereas the other 50 were hospitalized (Inpatients).

**Figure 1:**
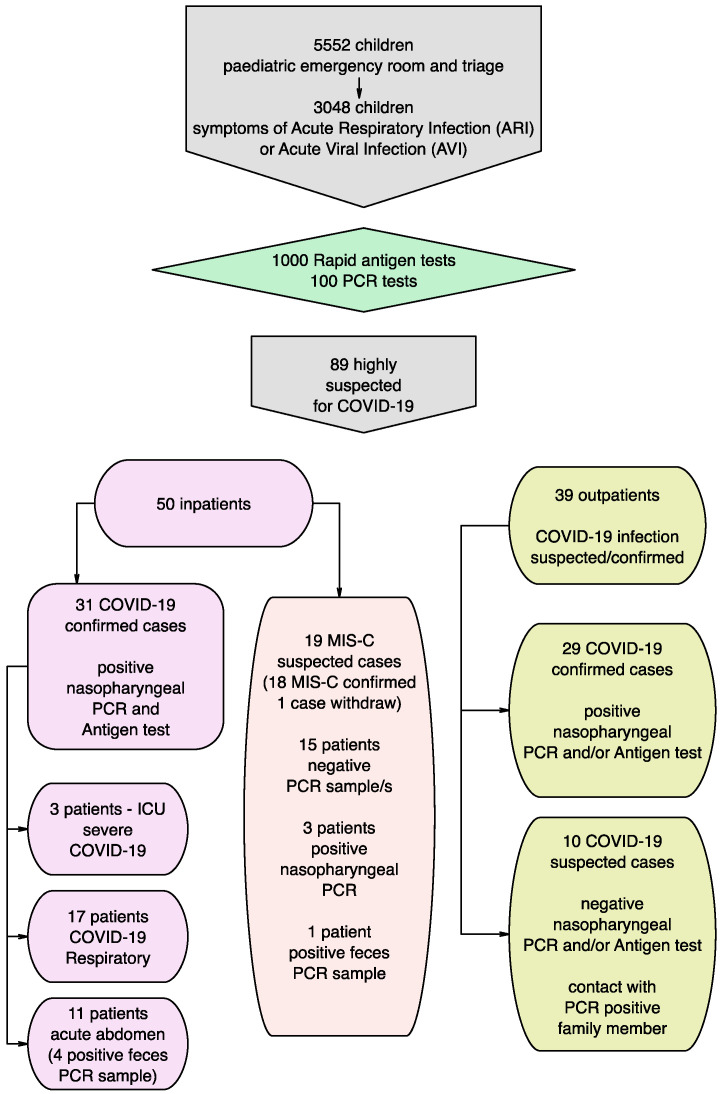
Study design—patient flows, groups and sub-groups.

As a result of 1000 SARS-CoV-2 rapid antigen tests and 100 PCR tests of nasopharyngeal swabs performed in highly suspected children (symptoms of acute respiratory infection (ARTI) and or acute viral infection (AVI)), COVID-19 infection was confirmed in 60 children (31 inpatients and 29 outpatients). In 10 children, the performed diagnostic test (PCR and/or antigen test) was negative, but there was a history for contact with a PCR-positive family member. In the pediatric COVID-19 ward, 31 of the children with PCR-confirmed active COVID-19 infection were hospitalized. We defined these cases as COVID-19 group with the following sub-groups: COVID-19 GI sub-group (with leading gastrointestinal symptoms); COVID-19 intensive care unit (ICU) sub-group (severe cases with the need for intensive care); and COVID-19 Respiratory sub-group (with predominant respiratory clinical manifestation). The design of the study is presented in Figure 1.

Nineteen children (*n* = 19) presented with symptoms meeting the criteria for MIS-C. Initially, negative PCR from the nasopharyngeal swab (before hospitalization) was diagnosed and treated in the general pediatric ward of the hospital. In addition, the children that met the following eligibility criteria for case definition for MIS-C [14] were included in the study and defined as MIS-C group:

1. Age under 18 with fever (>38.0 °C for ≥24 h or subjective fever lasting ≥24 h), inflammation (laboratory-confirmed by abnormal C-reactive protein (CRP), erythrocyte sedimentation rate (ESR), fibrinogen, procalcitonin (PCT), D-dimer, ferritin, lactic acid dehydrogenase (LDH), or interleukin 6 (IL-6), higher neutrophils, low lymphocytes, and low albumin) and clinically severe COVID-19 requiring hospitalization; presented with multisystem (>2) organ involvement AND
2. Other diagnoses excluded; AND
3. RT-PCR, antigen or serological tests proved recent or current infection with SARS-CoV-2; or exposure to the virus four weeks ago.

We considered MIS-C in any severe pediatric SARS-CoV-2 infection. Although the organ involvement usually consists of cardiac, renal, respiratory, hematologic, gastrointestinal, dermatologic, or neurological damage, liver failure can also be presented.

All children with MIS-C showed negative initial nasopharyngeal PCR and seropositivity, confirming passed SARS-Co-V-2 infection. However, repeated PCR during hospitalization showed a positive result in three cases—two from the nasopharyngeal specimen and one from feces.

### 2.2. Laboratory and Instrumental Testing of the Study Participants

The following investigations were applied to all of the children before their inclusion in the study: complete personal and family history, a physical examination from the multidisciplinary team—pediatricians and pediatric surgeons, PCR (nasopharyngeal swab) and/or antigen test prior to the hospitalization. In addition, in 15 children, repeated PCR (nasopharyngeal swab and or feces) were performed. Seventeen children were tested serologically for the presence of anti-SARS-CoV-2 antibodies (qualitative and quantitively determination of the serum IgM and IgG and or total IgM and IgG titer).

Routine microbiology testing (including hemoculture, urine culture, oropharyngeal swab), laboratory blood testing (complete blood count, CRP, procalcitonin, ESR, platelets, albumin, total protein, AST (Aspartate aminotransferase, SGOT), ALT (SGPT, Alanine aminotransferase), GGT (Gamma-glutamyl transferase), LDH (lactate dehydrogenase), ALP (Phosphatase, alkaline), total and direct bilirubin, hemostasis—INR (International Normalized Ratio), PT (Prothrombin Time) and urine analysis were performed. In moderate to severe cases, we performed CPK (Creatine phosphokinase), CK-MB fraction (creatine kinase-MB fraction), troponin I, fibrinogen, ferritin, and D-dimer testing. Additionally, serum levels of IL-6 were assessed by immunoassay in nine of the children in the MIS-C group (Elecsys, Roche diagnostics).

All children were examined ultrasonically (US), abdominal and thoracic; and fourteen with were examined with computer tomography (CT)—thorax alone in 5 children and the thorax/abdomen in 9 children. In addition, in all children, the heart function was evaluated with ECG (electrocardiography) records and 24-h vital signs monitoring.

The liver involvement evaluation in our study included the markers for hepatocellular injury (AST, ALT), cholestasis injury (GGT, ALP), bilirubin levels, markers for synthetic liver function (albumin and total protein) and hemostasis (INR, PT). Moreover, the observed biochemical indexes were interpreted according to age and sex, and the ULN/LLN are compliant with the pediatric reference values [15]. We defined the following two categories for liver involvement:

- Abnormal liver function—as any parameter (ALT, AST, ALP, GGT, and total bilirubin) more than the upper limit of the normal (ULN) lab reference value.
- Liver injury:
  ○ mild as ALT and/or AST over the ULN, but less than 2× ULN;
  ○ moderate 2–5× ULN;
  ○ severe > 5 times ULN, ALP, GGT, and/or total Bilirubin over 2× ULN.

We considered the physical examination as well—the palpable evaluation of the presence of hepatic and/or splenic enlargement (hepatosplenomegaly) and the US and/or CT liver and spleen dimensions, according to the age-adapted criteria.

### 2.3. Ethical Considerations

All parents signed their informed consent for the inclusion of their children in the study. Additionally, all children older than 12 years signed informed consent on their own before they participated in the study. The study was conducted following the Declaration of Helsinki. The Ethics Committee of the University Hospital “N. I. Pirogov.”approved the study design and protocol. 

### 2.4. Statistical Analysis and Data Handling

Statistical analysis of raw data was performed with SPSS^®^, IBM 2009, version 19 (2010), and Excel (v. 2010). We performed descriptive statistics, Kolmogorov–Smirnoff, T-tests, Mann–Whitney, ANOVA, χ2 or Fisher’s Exact test, correlation analysis. Significance level *p* < 0.05 was considered significant.

The study’s principal investigators performed data collection according to the hospital’s ethical and other policies and good clinical practice. The data were collected and coded uniformly to avoid any potential sources of bias. All efforts were made on the part of the authors to address potential bias. The statistical analysis was performed, omitting empty entries in the case of missing data (but no more than 5% of all the data entries). No sensitivity analyses were performed in our study.

## 3. Results

### 3.1. Demographic Characteristics and Epidemiological Data

In the Outpatient group, there were 29 confirmed cases with COVID-19 infection, and the other 10 were highly suspected (contact with PCR positive family member) with negative etiology diagnostic test. The children in this group had mean age 7.52 ± 2.30 (0–17 years), 52.2% were males and 47.8% were females, with leading symptoms of fever, cough, vomiting, diarrhea and abdominal pain. In addition, in 8 of 16 performed chest X-rays, we observed peri-bronchial thickening or interstitial infiltrates.

The mean age in the COVID-19 group was 7.54 ± 3.20 (0–17 years) with female predominance (40% male, 60% female). In the COVID-19 GI sub-group (11 children, 35%), the leading symptoms were gastrointestinal—eight children with acute abdomen (resulted in six appendectomies), one with mesenteric lymphadenitis, and two with gastroenteritis. The COVID-19 ICU sub-group included three children with severe COVID-19 infection and progressive ARDS, treated in the ICU, one of them a previously healthy 17-year-old girl with thrombotic complications, one case with partially corrected Fallot tetralogy (exitus letalis), and one case with concomitant Ohtahara syndrome. In the COVID-19 Respiratory sub-group—17 children with leading symptoms of acute respiratory tract infection (ARTI)—there were 10 children with moderate pneumonia, 3 with bronchitis, and 4 with upper airways infection. Four of the children with pneumonia had concomitant obesity.

The included children were from 1 to 17 years of age in the MIS-C group, at a mean age of 9.47 ± 3.79 years. Fifteen (78.9%) of the participants were males, and four (21.1%) were females. Only four (21.1%) out of nineteen children declared having had contact with an infected person prior to hospital admission; eight (41.1%) children had a history of recent COVID-19-suspected symptoms.

Two children fell within the 90th percentile of body mass index (BMI), i.e., slightly overweight, whereas all other children were between the 25th and 75th percentile. Only one child in this group had a chronic condition—mild cerebral palsy and epilepsy. The child had a minority ethnic background.

With the serology assessment of antibodies against SARS-CoV-2 testing, we found two children (10.5%) with positive class IgG, six (31.6%) with positive IgM, and two (10.5%) positive for both IgM and IgG antibodies.

The average hospital stay for all children was 8–9 days (ranging from 3 to 30 days).

### 3.2. Clinical Presentation of MIS-C Group

In the MIS-C group, days with fever ranged from 6 to 9. The maximal body temperature varied from 38 °C to 40.7 °C, where all children with MIS-C complained of fever (T > 38 °C). Eight children (42.1%) had a conjunctival injection, five (26.3%) had periorbital edema, and 10 (52.6%) had skin rash and/or mucosal enanthem. The skin rashes included palmar, plantar erythema, maculopapular, macular, and annular skin lesions.

The vast majority of the children (*n* = 16) complained of abdominal pain, and nine presented with severe acute abdomen. In addition, nine of the children with abdominal pain had co-existing ascites and US or CT data for enlarged mesenteric lymph nodes. The clinical symptoms of the MIS-C children are presented in Table 1.

Four (21.1%) children had a cough, four (21.1%) had ARDS, and six (31.6%) developed heart failure. Low blood pressure (as percentile for the age and height) was observed in five (26.3%) and high in two (10.5%) children. Five (26.3%) children needed oxygen administration. The mean SpO2 was estimated as 94.88 ± 4.05, ranging from 84 to 99.

We did not find significant differences between the clinical symptoms assessed in boys and girls.

### 3.3. Laboratory and Instrumental Findings

In four children (only 12%) from the COVID-19 group (except the ICU cases), we found elevated AST and/or ALT (up to 100). All of the children with elevated transaminase were overweight or obese, presented along with moderate COVID-19 pneumonia.

All ICU-treated children had slightly elevated AST/ALT at hospital admission. However, in the fatal outcome case only, we observed a tendency for gradual AST and ALT elevation (17–133–567 and 9–184–359, respectively) in parallel with the elevation of the inflammatory markers bilirubin and D-dimer (after the second week after disease onset). Thus, the laboratory parameters in this case resemble the typical MIS-C constellation.

The vast majority of the children in the COVID-19 group showed no significant liver involvement in contrast with the MIS-C group. Thus, we focused our analysis and observation on the MIS-C group.

Most of the pediatric MIS-C cases demonstrated mild to moderate leukocytosis (12.88 ± 5.9 × 10^9^/L) and lymphopenia (1.01 ± 0.72 × 10^9^/L). The average levels of routine laboratory testing are listed in Table 2. There were no significant differences between the levels assessed in boys and girls.

We found significantly higher levels of CRP, fibrinogen, ferritin, D-dimer and CPK, and significantly lower albumin levels and total serum protein in MIS-C compared to children with COVID-19. However, ALT, GGT, total and direct bilirubin were elevated in MIS-C, although without reaching statistical significance.

About half of the children in the MIS-C group (52%, *n* = 11) showed elevated transaminases (AST > 50 U/L). In eight patients, the AST level was >70 U/L. Nine children exerted ALT levels >50 U/L. The GGT was elevated in 10 cases, and in 6, the levels were hyper elevated (>100 U/L).

Elevated total bilirubin level >21 mmol/L was found in six children (in five of them, the AST/ALT was elevated). Direct bilirubin >3.5 mmol/L/was observed in eight children (in six cases, the elevation correlated with the higher level of total bilirubin). In more than half of the children (11 cases), the fibrinogen level was elevated (>4.5 U/L). Eight of ten children tested for ferritin levels showed elevated values (>200).

In most of the cases, the transaminases were tested two or three times during the hospital stay. Interestingly, the levels of AST/ALT during the admission and active treatment showed a decreasing, not increasing, tendency. On the other hand, we observed hypoalbuminemia in the vast majority of MIS-C children (73%, *n* = 14) in the initial blood testing and 89%, *n* = 17, in the repeated follow-up testing. In addition, the total protein was low in 68%, *n* = 13, of MIS-C children.

All of the children in the MIS-C group had elevated CRP (>5.0 g/mL). Significantly elevated PCT (>2.0) was documented in 17 cases (89%). Hyperinflammation state was one of the stable key laboratory characteristics of the MIS-C (as included in the diagnostic criteria). In 12 children (63%), we found elevated D-dimer level (>500 ng/mL), and in 8 cases (42%) the values were >1000 ng/mL.

In the MIS-C group with liver involvement (abnormal lung function or liver injury), we distinguished one outlier—the only child with comorbidities (cerebral palsy and epilepsy). In this case, we observed the most pronounced increase of the transaminases and development of thrombotic complication—occlusion of the hepatic veins (Budd Chiari). It remains debatable as to whether the child had MIS-C because of the lack of objectively proven fever, which is an obligatory diagnostic criterion. However, the other clinical, laboratory and imaging constellation indisputably points to the diagnosis of MIS-C with serological evidence for past SARS-CoV2 infection. In this child, we observed severe complications due to PIMS- TS/MIS-C post-COVID-19, with the need for liver transplantation. On the other hand, we could not exclude preexisting underlying liver disease in that child, considering that he took long-term anticonvulsants and had not been tested for thrombophilia before admission.

We did not find an association between the severity of the liver injury and adverse outcomes in the MIS-C group (except the Budd Chiari case) compared with the COVID-19 group. Instead, the liver involvement was correlated with severity (need for intensive care) and concomitant obesity. The other 18 children within the MIS-C group were previously healthy children without comorbidities, with BMI between the 25th and 75th percentile.

The majority of children with MIS-C showed pathological changes on their CT examinations, such as ground-glass opacity of the lungs (six children, 31.6%), posterior-basal consolidations (six children, 31.6%), and mesenteric lymphadenopathy (seven children, 36.8%). In seven out of nine children with abdominal CT, we observed enlarged liver size. In six of them, an enlarged spleen was observed. Upon physical examination, more than half of the children (*n* = 11) presented with hepatosplenomegaly.

In 11 children with MIS-C, we observed the US and/or CT data for pleural effusion, and in seven of them, ascites (or polyserositis). In six cases, we found a pericardial reaction with minimal pericardial effusion.

In two patients, CT data demonstrated sludge in the gallbladder, in the other two, thickening of the gallbladder wall. All of them had elevated AST (mean 362, min 81, max 1168) and ALT (mean 233.5, min 77, max 634), more than twofold elevated GGT (183.66 min 85, max 305), slightly elevated LDH (mean 457.5 min 246, max 975), elevated direct bilirubin (mean 7.45, min 4.9, max 10.6). One child presented with elevated total bilirubin and positive urobilinogen (1+). Pleural effusions and pericarditis were observed through US and/or CT examination in nine (47.4%) and five (26.5%) of the children, respectively.

The transaminases declined and normalized spontaneously in all patients without requiring any treatment, except the case with Budd Chiari complication.

Less is known regarding the association of coagulopathy with liver dysfunction in children with MIS-C in contrast to adult patients with COVID-19. Usually, INR is 1.0 for normal patients who are not on anticoagulation. In our patients, the coagulation tests were taken before the instigation of low molecular heparin treatment. Thirteen out of fifteen tested MIS-C children had INR between 1 and 1.5, while one had INR = 2.88 and one had INR = 0.87. Additionally, prothrombin time above 18 s was observed in 6/17 (35%) of MIS-C children.

Additionally, in three cases, microbiology testing revealed different pathogenic microorganisms in the nasopharyngeal swab (i.e., *Streptococcus pneumoniae*, *Staphylococcus aureus*, *E.coli*). All other microbiologically tested specimens in the MIS-C group were sterile or showed normal microflora. Four children with cervical lymph adenomegaly were tested serologically (for anti-EBV-VCA-IgM and IgG antibodies) to exclude alternative diagnoses. All of these tests were negative.

### 3.4. Clinical–Laboratory Correlations and Observations

In addition to the aforementioned main clinical presentation and laboratory and instrumental findings, we found the following clinical correlations.

We found higher IL-6 levels in children with positive PCR from nasopharyngeal swab (71.72 ± 21.72 vs. 62.46 ± 10.11, *p* = 0.008), and decreased SpO2 (94.50 ± 4.58 vs. 95.00 ± 1.00, *p* = 0.019). Additionally, lower fibrinogen levels were observed in children that had symptoms of COVID-19 4–6 weeks before hospital admission (4.06 ± 1.71 vs. 6.00 ± 1.15, *p* = 0.045).

**Figure 2:**
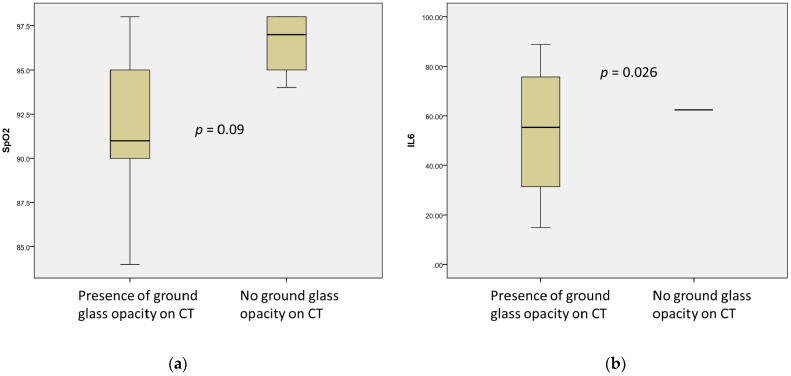
Association of ground-glass opacity (CT of lungs) with Sp02 ((**a**), *n* = 12) and IL-6 ((**b**), *n* = 9) levels.

Ground-glass opacity was also associated with lower levels of IL-6 (53.58 ± 30.85 ng/mL vs. 62.46 ± 28.12 ng/mL, *p* = 0.026) and decreased levels of SpO2 (91.5 ± 4.86 vs. 96.5 ± 1.92, without reaching significance, *p* = 0.09) (Figure 2). Moreover, ground-glass opacity and reduced SpO2 were moderately correlated (r = 0.567, *p* = 0.87).

The presence of consolidations was found more frequently in patients with lower levels of GGT (122.66 ± 53.84 vs. 416.00 ± 93.25, *p* = 0.016) and fibrinogen (3.03 ± 1.44 vs. 5.30 ± 1.13, *p* = 0.044). In contrast, mesenteric lymphadenitis was observed more often in patients with elevated LDH (327.83 ± 159.39 vs. 281.00 ± 38.97, *p* = 0.077). No associations were found for pleural effusions, unlike pericarditis. The latter was more frequent in patients with lymphopenia (1.03 ± 0.62 vs. 0.48 ± 0.19, *p* = 0.07), higher procalcitonin (22.77 ± 10.14 vs. 6.69 ± 3.45, *p* = 0.082), higher CPK (618.00 ± 382.00 vs. 86.33 ± 38.75, *p* = 0.079), and decreased albumin (26.00 ± 6.82 vs. 33.18 ± 1.23, *p* = 0.057).

**Figure 3:**
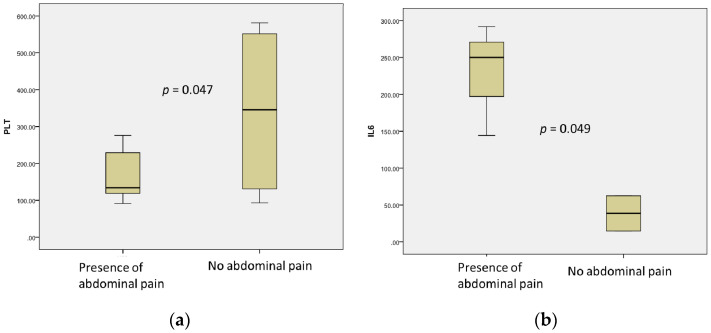
Association of abdominal pain with low platelet counts (**a**), *n* = 19; and elevated levels of IL-6 (**b**), *n* = 9.

Abdominal pain was observed mainly in patients with low platelet count (164.00 ± 64.85 vs. 341.25 ± 122.96, *p* = 0.047) and total levels of proteins (62.60 ± 7.37 vs. 53.75 ± 3.92, *p* = 0.069) and higher IL-6 levels (228.70 ± 75.99 vs. 38.67 ± 23.79, *p* = 0.049) (Figure 3).

**Figure 4:**
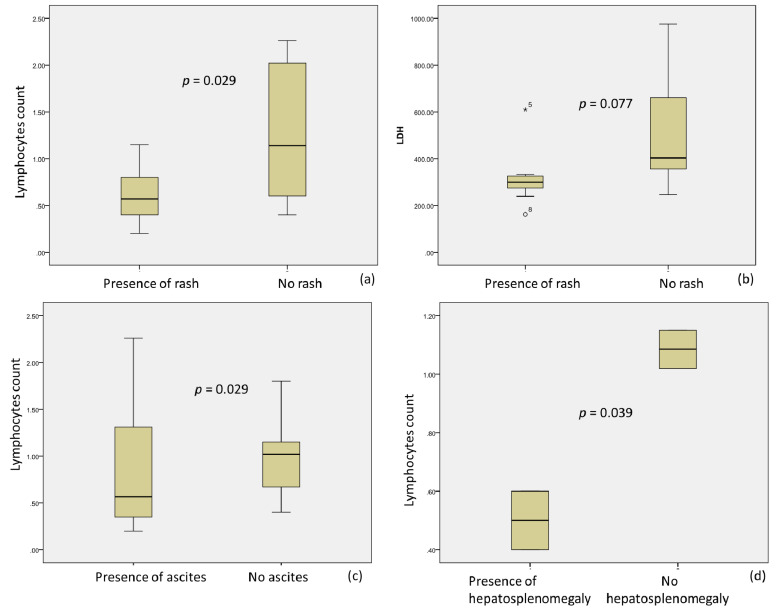
Association of rash with lymphopenia (**a**) and LDH levels (**b**), as well as ascites with lymphopenia (**c**) and hepatosplenomegaly with lymphopenia (**d**). *5 and °8 (**b**) denote outliers.

Conjunctival injections and periorbital edema correlated with elevated GGT levels (222.00 ± 10.43 vs. 33.33 ± 19.76, *p* = 0.014) and maximal fever (38.00 vs. 39.00 *p* = 0.022). Moreover, skin rash was observed more frequently in patients with a lack of lymphopenia (0.62 ± 0.30 vs. 1.26 ± 0.74, *p* = 0.029) and a low level of LDH (316.55 ± 122.31 vs. 528.20 ± 130.81, *p* = 0.077) (Figure 4a,b).

Ascites was associated with lymphopenia (0.86 ± 0.80 vs. 0.96 ± 0.42, *p* = 0.029) (Figure 4c) and elevated LDH (464.42 ± 110.83 vs. 311.50 ± 54.38, 0.077). Hepato-splenomegalia was also found more frequently in children with lymphopenia (0.5 ± 0.14 vs. 1.09 ± 0.9, *p* = 0.039) (Figure 4d), higher troponin I (402.00 ± 101.23 vs. 3.00 ± 2.12, *p* = 0.004), and low ESR (5.00 vs. 17.00 ± 1.41, *p* = 0.091).

Five children had US evidence for enlarged mesenteric lymph nodes, nine had pleural effusion, and five pericardial reactions with small effusion. No correlation of serositis with hypoalbuminemia or other laboratory parameters was demonstrated. Three children had US, laboratory, ECG, and clinical evidence for myocardial dysfunction.

We also found that lymphopenia correlated with AST (r = 0.523, *p* = 0.031) and the presence of rash (r = 0.546, *p* = 0.29) and hepato-splenomegaly (r = 0.961, *p* = 0.039). ALT, AST, GGT and LDH were correlated, as well (*p* < 0.05). The levels of IL-6 were correlated inversely with abdominal pain (r = −0.880, *p* = 0.049) and the presence of pleural effusions (r = 0.710, *p* = 0.049). There was no correlation between abdominal pain and low protein levels, although there was a tendency for such (r = −0.421, *p* = 0.069). No association was documented between abdominal pain and the presence of ARSD.

CRP was correlated with the presence of diarrhea and need for human serum albumin administration (r = 0.502, *p* = 0.040; r = 0.601, *p* = 0.039, respectively). The presence of diarrhea was a more frequent symptom in patients with lower CRP (9.00 ± 3.44 vs. 22.25 ± 2.58, *p* = 0.04), but higher AST and ALT (469.00 ± 349.59 vs. 48.50 ± 8.98, and 286.67 ± 174.91 vs. 57.78 ± 15.47, respectively, *p* = 0.010), and higher D-dimer (4516.66 ± 715.83 vs. 1066.33 ± 356.29, *p* = 0.001).

**Figure 5:**
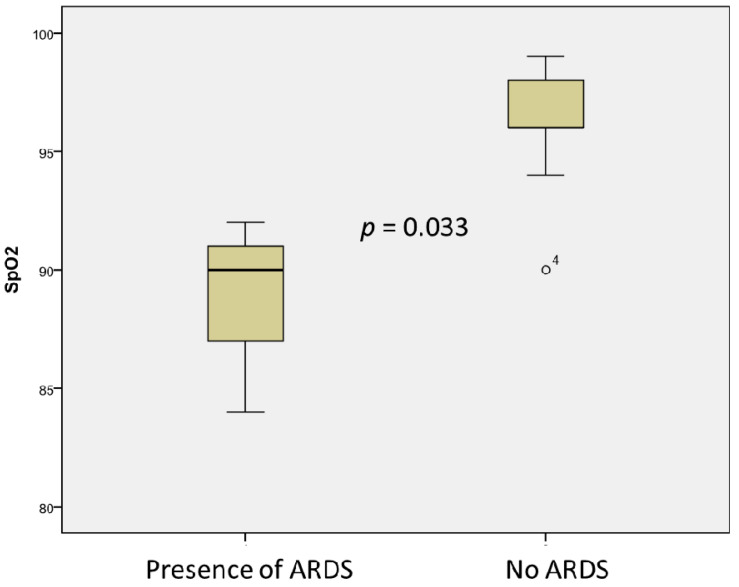
Association of SpO2 with the presence of ARDS in children with MIS-C. °4 denotes an outlier.

Cough was associated with lower albumin levels (22.66 ± 1.52 vs. 32.64 ± 1.23, *p* = 0.003) and total serum protein levels (45.66 ± 2.5 vs. 60.64 ± 7.49, *p* = 0.004), as well as low saturation (91.25 ± 6.18 vs. 96.08 ± 2.35, *p* = 0.033). ARDS was associated with low albumin (23.00 ± 1.41 vs. 33.31 ± 4.06, *p* = 0.003), total serum protein levels (48.00 ± 5.09 vs. 61.08, *p* = 0.004), and oxygen saturation in the blood (88.67 ± 4.16 vs. 96.31 ± 2.39, *p* = 0.033) (Figure 5). A non-significant association was observed for the presence of heart failure and high fibrinogen levels (6.23 ± 1.12 vs. 4.46 ± 1.59, *p* = 0.074) in children with MIS-C.

### 3.5. Therapy Administration

Standard therapy for COVID-19 was administered, adjusted for the individual presentation of the disease. Thirteen children received low-molecular-weight heparin (LMWH—Enoxaparin sodium, Clexan); five of them continued with aminosalicylic acid one month after discharge. In nine children, human serum albumin was administered; in five, intravenous immunoglobulin (IVIG); five children received oxygen supply (nasal cannula or facial mask), while one was on nasal CPAP. In addition, all children had antibiotic therapy (meropenem, amikacin, ceftriaxone, amoxicillin, cefuroxime, etc., as monotherapy or combinations of antibiotics), systemic corticosteroids (methylprednisolone), fluid therapy, NSAIDs, probiotics, and antimycotics.

## 4. Discussion

While SARS-CoV-2 has been implicated in causing liver damage by binding to the ACE-2 receptors in bile duct epithelial cells [16]. This has not been scientifically proven in children to date. The observed liver damage in uncomplicated COVID-19 cases is mainly temporary and usually resolves without treatment [17].

During the fall and winter months of 2020 and the first three months of 2021, 19 children were hospitalized in the pediatric department of University Hospital “N. I. Pirogov”, Sofia, and were found to meet the diagnostic criteria for MIS-C. Most of them exhibited gastrointestinal symptoms, some to the point of imitating acute abdomen, accompanied by highly elevated inflammatory markers—several children presented with elevated liver enzymes, as well as hypoproteinemia and hypoalbuminemia. A decrease of the enzymes and liver-dependent inflammatory markers was later observed, probably due to recovering. Radiological methods demonstrated lung consolidation with pleural effusions, cardiac involvement, mesenteric lymphadenopathy, and overall enteric and bowel involvement in some patients.

In a study dating from September 2020, Zhou et al. showed that liver involvement in COVID-19 was found to affect mainly those aged 0–3 years compared to older children. Liver immaturity at this young age is the suspected reason [18]. As far as the liver enzymes are concerned, pediatric patients often present with elevated levels at admission, in contrast to adults, whose enzyme levels usually rise no sooner than their second week of hospital stay [19]. Our study found similar observations, more widely demonstrated in the MIS-C group than in the COVID-19 group.

Most documented MIS-C cases have shown positive SARS-CoV-2 antibodies (87%) and less frequently positive RT-PCR from nasopharyngeal swabs (32%). Thus, this condition might be post-infectious rather than connected to acute early infection. It is now accepted that MIS-C is an inflammatory immunological condition linked with previous symptomatic or asymptomatic COVID-19 infection [20].

In our MIS-C group, only two children were PCR positive from on the basis of repeated nasopharyngeal specimen collection and one on the basis of feces specimen during the hospital stay after the initial negative test results.

Even though the etiological cause of the COVID-19 and MIS-C is probably the same, a distinction should be made between the liver injury seen in acute SARS-CoV-2 infection and the one in MIS-C due to separate mechanisms of liver involvement [12]. In acute COVID-19 infection, some signs suggest direct liver damage from hepatotropic viral invasion due to the mentioned ACE2 receptors found on the surface of the liver and bile duct epithelial cells [3]. In contrast, there is a precise immune-mediated mechanism of hepatotoxicity in MIS-C [21]. On the other hand, liver involvement was observed in severe COVID-19 infection by developing the notorious “cytokine storm” and multiorgan impairment, where cytokines promote liver enzymes’ release and elevation [22]. The development of the seen changes is similar to sepsis-associated liver dysfunction: elevated liver markers, elevated bilirubin concentration, impaired synthesis function resulting in hypoproteinemia and coagulation disorders [22].

IL-6 involvement in liver failure during COVID-19 is also established as a part of the cytokine storm [4]. In some cases, abdominal pain is due to mesenteric lymphadenitis, which might also be connected with IL-6 [23]. In adults, a link between IL-6 and ARDS severity was found [1], but no such correlation was documented in our study. We found that abdominal pain was associated with higher IL-6 levels along with decreased platelet counts. The observation that abdominal pain and thrombocytopenia correlated is probably because of the leading abdominal form of the infection.

Despite the small number of children with positive PCR (nasopharyngeal or fecal), which could be the cause of bias, we found a link between the persistence of the virus, its slower clearance, and more significant cytokine activation, demonstrated by elevated IL-6.

Such cytokine network disruption is also observed in MIS-C [24]. The difference with the cytokine storm observed in adults is that it occurs with a latent period after acute infection, which may be due to both delayed clearance of the virus (mainly in the gastrointestinal tract) or involvement of purely immunological mechanisms. In adults, the last immunological phase also occurs after the acute phase (usually after ten days) [4]. In children, due to the frequent asymptomatic course, it is difficult to assess when the infection began. Therefore, it is challenging to define the exact time window for the onset of MIS-C symptoms. However, we can hypothesize that the children can start the infection’s immunological phase after a more extended period. In line with this, MIS-C in children may resemble severe COVID-19 with cytokine storm in adults.

This is also confirmed by our results, as 100% of children with COVID-19 treated at ICU have elevated transaminases, similar to the observations in adults with the severe course. Conversely, in the group of hospitalized children without the need for intensive care, only 12% show slightly elevated liver enzymes. All of the latter children were with obesity. Conversely, the picture of MIS-C indicates slightly more than half of children showing liver involvement. This observation was reported in the study of Perez e al., which compared liver involvement in children with MIS-C and COVID-19. The authors demonstrated that children with MIS-C had 2.3× increased odds of elevated ALT levels compared to children with COVID-19 after adjusting for age and race. [25].

The data showed that aminotransferases and bilirubin levels are mainly affected in severe COVID-19 patients with multiorgan failure [26]. Of our MIS-C group, five children had slightly elevated total and indirect bilirubin, and eleven (52%) had elevated transaminase—AST >50 U/L. In eight patients, the AST level was >70 U/L. Nine children had ALT levels >50 U/L. The GGT was elevated in 10 cases (52%), and in sic cases (31%), it was >100 U/L. Perez et al. reported similar data: 36% cholestasis in their cohort of children with liver involvement. Interestingly, the elevated liver enzymes correlated with the presence of diarrhea, elevated D-dimer and lower CRP. Thus, the lower level of CRP (but elevated procalcitonin) in this patient group could be explained by decreased synthetic liver function and elevated D-dimer with hypercoagulation (or hyper coagulopathy).

In our MIS-C group, 86.6% of children had INR between 1 and 1.5, and two children had INR below 1 or above 2.5. Additionally, prolonged PT was observed in 35% of MIS-C children. However, our patients showed no clinical signs and were not “auto-anticoagulated”, even with high INR or prolonged PT. Therefore, the higher INR/PT ratio might reflect homeostatic coagulation anomalies and therefore increase thrombotic risk. This corresponds to the increased D-dimer demonstrating hyperactivated coagulation/fibrinolysis.

Another critical point in liver damage seen in COVID-19 infection is drug-induced hepatotoxicity caused by the multidrug management of both acute infection and MIS-C [27]. We could not find a connection between the specific medications and liver injury in the MIS-C group. Before the hospitalizations and baseline blood test, all children were treated with conventional medications and dosages (short courses of NSAIDs and antibiotics). During the hospital stay, in all cases (including the Budd Chiari patient), we observed a tendency for liver enzymes to decline and normalize, rather than the opposite. None of our patients (COVID-19 inpatient/outpatient and MIS-C) were treated with antiviral or hepatoprotective medications.

As per the gathered clinical data, childhood obesity is a substantial risk factor for a more severe clinical presentation of COVID-19 infection with severe pneumonia and a need for support [28]. On the other hand, literature data show that the liver involvement seen in acute SARS-CoV-2 infection is linked to preexisting fatty liver disease (nonalcoholic fatty liver disease NAFLD) in children with higher BMI than that seen in MIS-C, which coincides with our observation as well [29]. Our observations also confirmed this: all of the children in the COVID-19 group (except the ICU cases) with elevated transaminase were overweight or obese with moderate COVID-19 pneumonia. In these children, we could not exclude the obesity influence in the pathogenesis of liver enzyme elevation [30].

Multiple studies demonstrate that most children diagnosed with COVID-19 have little or no complications during acute infection. Therefore, supportive care in pediatric COVID-19 cases has been recognized as the mainstay in management strategy [26].

Managing MIS-C is a different matter, considering the various differential diagnoses and clinical presentation patterns and evolution. No specific antiviral medications for COVID-19 have to date proved to be helpful. In more critical cases, complex therapy, including human serum albumin, glucocorticoids, diuretics, antibiotics, and immunoglobulins, was initiated with an overall pleasing effect. Managing MIS-C was made possible due to close communication of a multidisciplinary team of pediatricians, pediatric surgeons, and radiologists.

Intravenous immunoglobulins (IVIG) and glucocorticoids are widely used as immunomodulatory treatments when clinical deterioration is established. Our team has also followed suit. Furthermore, there is evidence that Anakinra (>4 mg/kg/day IV or SC) may effectively be used as an immunomodulator in pediatric COVID-19 patients with associated hyper inflammation when IVIG and glucocorticoids fail, especially when implemented at the first signs of clinical deterioration [31].

We treated only the severe cases of MIS-C with human serum albumin. Protein losses (requiring human serum albumin treatment) could be due to polyserositis and interstitial losses (due to increased permeability of the serous membranes) or perhaps enteritis losses. However, this question has not yet been answered in the literature. None of the children had significant proteinuria, which could explain potential urine protein losses. Due to abdominal pain, children often refuse to eat, but the alimentary component itself could only partially explain hypoalbuminemia. We suggest that this could be explained by reduced synthetic liver function. In turn, hypoalbuminemia aggravates the condition in children because it leads to reduced intravascular volume, and hence reduces diuresis, leading to hypotension and edema.

Usually, diarrhea is associated with both liver involvement and hypercoagulable states [32]. Additionally, diarrhea as a symptom may be due to local infectious or immune inflammation (such as enteritis), as well as the result of polyserositis. Therefore, there might be a possible link between enteritis and hepatitis (or liver dysfunction/damage). We did not find a correlation between the presence of mesenteric lymphadenitis and diarrhea and the levels of INR and D-dimer in our MIS-C patients.

An Italian multicenter study suggested that preexisting chronic liver disease in children is not an additional risk factor to be considered in severe COVID-19 infection development [21]. However, the data on COVID-19 in pediatric patients with liver diseases is still not sufficient, especially for pediatric liver transplant patients [33]. Nevertheless, with the exhaustion of healthcare systems globally during the pandemic, many patients with underlying chronic illnesses have been deprived of the necessary monitoring and management, in many cases with delayed appointments, tests, and therapeutic interventions. In this sense, in a purely logistical matter, the COVID-19 pandemic has proven harmful to many children with chronic liver conditions. Furthermore, it was shown that liver damage prior to COVID-19 infection could be an independent prognostic factor [34,35].

The long-term effects of the SARS-CoV-2 virus on the liver, whether from acute COVID-19 or MIS-C, are yet to be observed. Therefore, the recommendations indicate that it is necessary to continue to evaluate liver enzymes weeks after discharge in an outpatient setting to prevent any continuing liver damage [29]. Unfortunately, this type of follow-up is only the tip of the iceberg, as only some of the children are diagnosed with SARS-CoV-2 infection and will be appropriately monitored. Nevertheless, there is a marked need for post-COVID follow-up and rehabilitation protocols regarding liver function and neurological, respiratory, and mental support [36].

In all our MIS-C patients, the disease ended with complete recovery. One patient had MRI data for myocardial infarction (but the child recovered). Unfortunately, one child developed a complication—hepatic vein thrombosis (Budd Chiari syndrome). The latter underwent successful liver transplantation. In the COVID-19 group, we observed complications only in the ICU group—one exitus letalis and one thrombotic complication (post-stroke hemiparesis). All other children were followed up (at between 1 and 3 months). We did not record any complaints or permanent disabilities and objectified complete normalization of paraclinical parameters.

Our study has limitations, such as the relatively small number of children with MIS-C, and the results being reported from a single center. However, considering the relatively low incidence of MIS-C, our data on these children are significant and can be clinically helpful. It is possible that some of the correlations should be omitted because of the small subgroups. Additionally, the included children and their monitoring were observed only during the third epidemic (and the most serious so far) wave in the capital city. Therefore, the study is single-centered, and the impact of new strains of the virus cannot be assessed. Another limitation is the severity of the disease in the COVID-19 group—only three children had a severe course requiring intensive care. Most had a mild to moderate course, with rare involvement of the liver and liver failure, respectively. Due to the milder course of infection, only some of the children in the COVID-19 group underwent more extensive biochemical studies, which limited the possibility for more detailed multivariable analysis.

However, our study’s strengths include the thorough investigation and description of our patients and the clinically valid results obtained with respect to liver involvement during MIS-C and COVID-19. Studies on children with COVID-19 and MIS-C are scarce, especially those investigating specific organ involvement, i.e., the liver. Therefore, we believe that our observations and recommendations will be helpful for pediatricians and other clinicians’ practices.

## 5. Conclusions

The presented data demonstrate liver involvement in MIS-C, drawing some valuable conclusions and recommendations for the clinical practice of pediatricians. The liver can also be involved in MIS-C, with typical laboratory and instrumental outcomes. However, the clinical course of MIS-C with abdominal pain and pronounced inflammatory activity without a specified focus and causative agent may mimic the picture of acute abdomen (acute appendicitis, peritonitis, complicated mesenteric lymphadenitis). Additionally, the long-term effects of the SARS-CoV-2 virus on the liver, whether from acute COVID-19 or MIS-C, are yet to be observed. Thus, the clinical course of MIS-C, with abdominal pain and pronounced inflammatory activity without a specified focus and causative agent, can be unpredictable, and presents a challenge with respect to proper management.

The recommendations indicate that it is necessary to continue evaluating liver enzymes weeks after discharge in an outpatient setting to prevent any continuing liver damage or exclude preexisting undiagnosed chronic liver pathology. Unfortunately, this type of follow-up is only the tip of the iceberg. Only some of the children will be diagnosed with SARS-CoV-2 infection and will be appropriately monitored. Nevertheless, there is a marked need for post-COVID follow-up and rehabilitation protocols regarding liver function, as well as neurological, respiratory, and mental support.

## Figures and Tables

**Table 1:** Leading symptoms in MIS-C group of children and their distribution according to gender.

Symptoms	Male (*n*, %)	Female (*n*, %)	All (*n*, %)
Fever > 38 °C	13 (86.7%)	4 (100%)	17 (89.5%)
Days with fever > 5 days	15 (100%)	4 (100%)	19 (100%)
Exanthema	8 (53.3%)	3 (75%)	11 (57.9%)
Abdominal pain	13 (86.7%)	3 (75%)	16 (84.2%)
Acute abdomen	8 (53.3%)	2 (50%)	10 (52.6%)
Vomiting	4 (26.7%)	1 (25%)	5 (26.3%)
Diarrhea	7 (46.7%)	0	7 (36.8%)
Periorbital oedema	9 (60.0%)	4 (100%)	13 (68.4%)
Peripheral oedema	8 (53.3%)	4 (100%)	12 (63.2%)
Icterus	13 (86.7%)	4 (100%)	17 (89.5%)
Meningeal/radicular syndrome	4 (26.7%)	0	4 (21.1%)
Conjunctival injection	10 (66.7%)	2 (50%)	12 (63.2%)
Cough	4 (26.7%)	0	4 (21.1%)
Respiratory distress, ARDS	4 (26.7%)	0	4 (21.1%)

**Table 2:** Comparison of laboratory findings in children with COVID-19 infection and those with MIS-C. Data are presented as mean ± SD.

Laboratory Parameter, Unit, ULN	COVID-19	MIS-C	Significance, *p*
AST, ULN * U/L	46.75 ± 12.58	122.71 ± 65.99	0.168
ALT, ALN * U/L	26.40 ± 6.81	98.18 ± 36.21	0.054
GGT, ULN * U/L	50.77 ± 18.87	118.2 ± 31.37	0.080
ALP, ULN * U/L	139.40 ± 45.05	135.45 ± 19.25	0.925
LDH, ULN 248 U/L	269.40 ± 50.41	382.87 ± 54.51	0.133
ESR, ULN 12 mm	31.50 ± 8.83	33.06 ± 5.37	0.867
CRP, ULN 0.5 mg/dL	3.54 ± 1.07	19.13 ± 10.5	<0.001
PCT, ** ng/mL	16.14 ± 15.18	10.33 ± 3.09	0.653
Fibrinogen, ULN 4.5 g/L	3.6 ± 0.27	5.01 ± 0.45	0.007
Ferritin, ULN f 120, m 250 * ng/mL	136.40 ± 47.09	591.93 ± 136.13	0.003
Amylase, ULN 100 U/L	39.00 ± 4.60	29.7 (10–47)	0.166
Lipase, ULN 140 U/L	23	20.1 (4.7–47.2)	0.880
Bilirubin total, ULN 21 * umol/mL	9.77 ± 2.70	22.9 ± 6.57	0.092
Bilirubin direct, ULN 2 * umol/mL	2.39 ± 0.5	11.39 ± 4.81	0.071
Albumin, LLN 36 * g/L	39.06 ± 1/65	30.88 ± 5.75	0.046
Total Serum Protein, LLN 66 * g/L	65.64 ± 2.13	58 ± 9.00	0.031
D-dimer, ULN old 100, new 500 ngFEU/mL	540.08 ± 236.06	1928 ± 543.56	0.037
CPK, ULN m 171, f 145 U/L	73.00 ± 23.45	201 ± 104.40	0.001
CPK-MB, N 0.6–6.3 ng/mL	N/A	2.9 ± 1.44	N/A
Hs troponin I, ULN m 19.8, f 11.6 pg/mL	N/A	110.83 ± 64.05	N/A
Interleukin 6, ULN-6 ng/mL ***	N/A	120.36 ± 35.56	N/A

* Reference ranges according to age and sex—Albumin 1–3 years (34–42 g/dL), 4–19 years (35–56 g/dL), Total Protein 1–7 years—61–79 g/L, 8–12 years—64–81, 13–19 years—66–82, GGT 4 months-10 years—5–32 U/L, ALT (SGPT) Alanine aminotransferase—1–19 years—5–45 U/L, AST (SGOT) Aspartate aminotransferase—1–3 years—20–60 U/L, 3–9 years—15–50, 10–15 years—10–40, 16–19 years—15–45, LDH (lactate dehydrogenase) 1–9 years—150–500 U/L, 10–19 years—120–330 U/L, ALP (Phosphatase, alkaline)—1–9 years 145–420 U/L, 10–11 years (240–560), 12–13 years male 200–495, female 105–420. 14–15 years male 130–525, female 70–230, 16–19 years male 65–260, female 50–130, Ferritin 1–9 years—10–60 mg/L, 10–18 years—male—10–300, female 10–70. ** PCT interpretation (<0.50—low risk, 0.5–2.0—follow up, >2.0—high risk) ng/mL. *** in 9 children.

## Data Availability

The data presented in this study are available on request from the corresponding author. The data are not publicly available due to restrictions, e.g., privacy or ethical.
